# Subretinal gene delivery using helper-dependent adenoviral vectors

**DOI:** 10.1186/2045-3701-1-15

**Published:** 2011-04-04

**Authors:** Linda Wu, Simon Lam, Huibi Cao, Rui Guan, Rongqi Duan, Derek van der Kooy, Rod Bremner, Robert S Molday, Jim Hu

**Affiliations:** 1 Physiology and Experimental Medicine Program, Hospital for Sick Children, 555 University Avenue, Toronto, Ontario, M5G1X8, Canada; 2 Department of Laboratory Medicine and Pathobiology, University of Toronto, 1 King's College Circle, Toronto, Ontario, M5S1A8, Canada; 3 Department of Paediatrics, University of Toronto, 555 University Avenue, Toronto, Ontario, M5G1X8, Canada; 4 Department of Molecular Genetics, University of Toronto, 1 King's College Circle, Toronto, Ontario, M5S1A8, Canada; 5 Toronto Western Research Institute, University Health Network, University of Toronto, 399 Bathurst Street, Toronto, Ontario, M5T 2S8, Canada; 6 Department of Biochemistry and Molecular Biology, 2350 Health Sciences Mall, University of British Columbia, Vancouver, B.C. V6T 1Z3, Canada

## Abstract

This study describes the successful delivery of helper-dependent adenoviral vectors to the mouse retina with long term and robust levels of reporter expression in the retina without apparent adverse effects. Since these vectors have a large cloning capacity, they have great potential to extend the success of gene therapy achieved using the adeno-associated viral vector.

## Background

The eye has several unique features that make it a well suited target organ for gene therapy. It has a highly compartmentalised structure which allows for the efficient delivery of a small volume of vector suspension to a specific subset of cells. The precise targeting of a particular cell type minimises viral dissemination and unwanted systemic effects. Additionally, immune responses resulting from intraocular administration are attenuated compared to those following systemic administration because the eye has both physical barriers as well as an internal environment that promotes tissue preservation and protects against harmful inflammatory responses that can limit transgene expression. Lastly, a wide range of well characterized animal models are available for studies of eye disease progression [1,2].

Several recent gene therapy trials have brought clinical benefits to patients with Leber's Congenital Amaurosis, a severe childhood retinal dystrophy [3-6]. Currently, the majority of eye gene therapy trials are carried out by using the adeno-associated virus (AAV). Although earlier work with AAV was shown a lag between viral vector injection and transgene expression, the self-complementary AAV (scAAV) has been shown to express the transgene in as little as one-day after injection, and can transfect the photoreceptor cells in addition to the RPE [7]. However, the limited cloning capacity of the AAV vector (4.7 kb) [8] is a major obstacle to delivery of large therapeutic genes or genes with long DNA regulatory elements that has yet to be overcome. Although some have attempted to deliver large genes using AAV[9,10], the latest studies have revealed that the expression of the transgenes was a result of co-infection and recombination within target cells [11]. The existence of retinal diseases involving genes beyond the AAV's cloning capacity encourages studies of potential viral gene therapy vectors beyond AAV. For example, *ABCA4 *is a member of the ATP-binding cassette transporter sub-family, mutations of which are linked to Stargardt macular dystrophy. As another example, *CEP290*, a gene encoding a 290 kDa centrosomal protein, is associated with a frequent form of Leber's Congenital Amaurosis (LCA10). The cDNAs of these genes are 6.8 kb and 7.4 kb respectively, precluding the use of AAV vectors even before consideration of regulatory elements. Thus, it is important to develop alternative vectors that have a large cloning capacity, the ability to transduce non-dividing cells in the post-mitotic retina, and a low immunogenicity to allow sustained long-term transgene expression. The helper-dependent adenoviral (HD-Ad) vector presents all these characteristics which make it an ideal candidate for retinal gene therapy.

HD-Ad vector, also known as the gutless, gutted, or high-capacity Ad vector, has been developed with significant improvements in the safety and delivery efficiency after many changes made to the first generation adenoviral (FG-Ad) vectors [12-16]. The main difference in genome composition between the HD-Ad vector and its parental FG-Ad vector is that the HD-Ad vector is fully devoid of all viral coding genes, leaving only the ITR and ψ sequences necessary for vector replication and packaging, respectively [17,18]. This strategy prevents the production of any viral proteins which in turn significantly reduces the cytotoxic T lymphocyte (CTL) response brought upon by viral gene expression [19-21]. A minimized immune response reduces toxicity to host cells, delays vector clearance, and promotes long-term transgene expression. In fact, upon HD-Ad injection through the tail vein of mice, transgene expression in livers has been shown to have life-long persistence [22]. However, very little is known about the utility of HD-Ad vectors in retinal gene therapy [23,24], especially regarding the delivery of HD-Ad into the subretinal space[19,25]. We hypothesized that the HD-Ad system can be used to deliver transgenes into retinal pigment epithelial (RPE) and photoreceptor (PR) cells.

We performed extensive analyses of HD-Ad vector delivery to mouse subretinal space using LacZ as a reporter gene and found that the HD-Ad elicits transgene expression for a minimum of 2 months with no sign of decrease in expression. We also observed a dose response in reporter gene expression. Our results show that HD-Ad vectors have great potential to extend the success of eye gene therapy to applications which require vectors for delivering large genes or regulatory elements.

## Results and discussion

**Figure 1:**
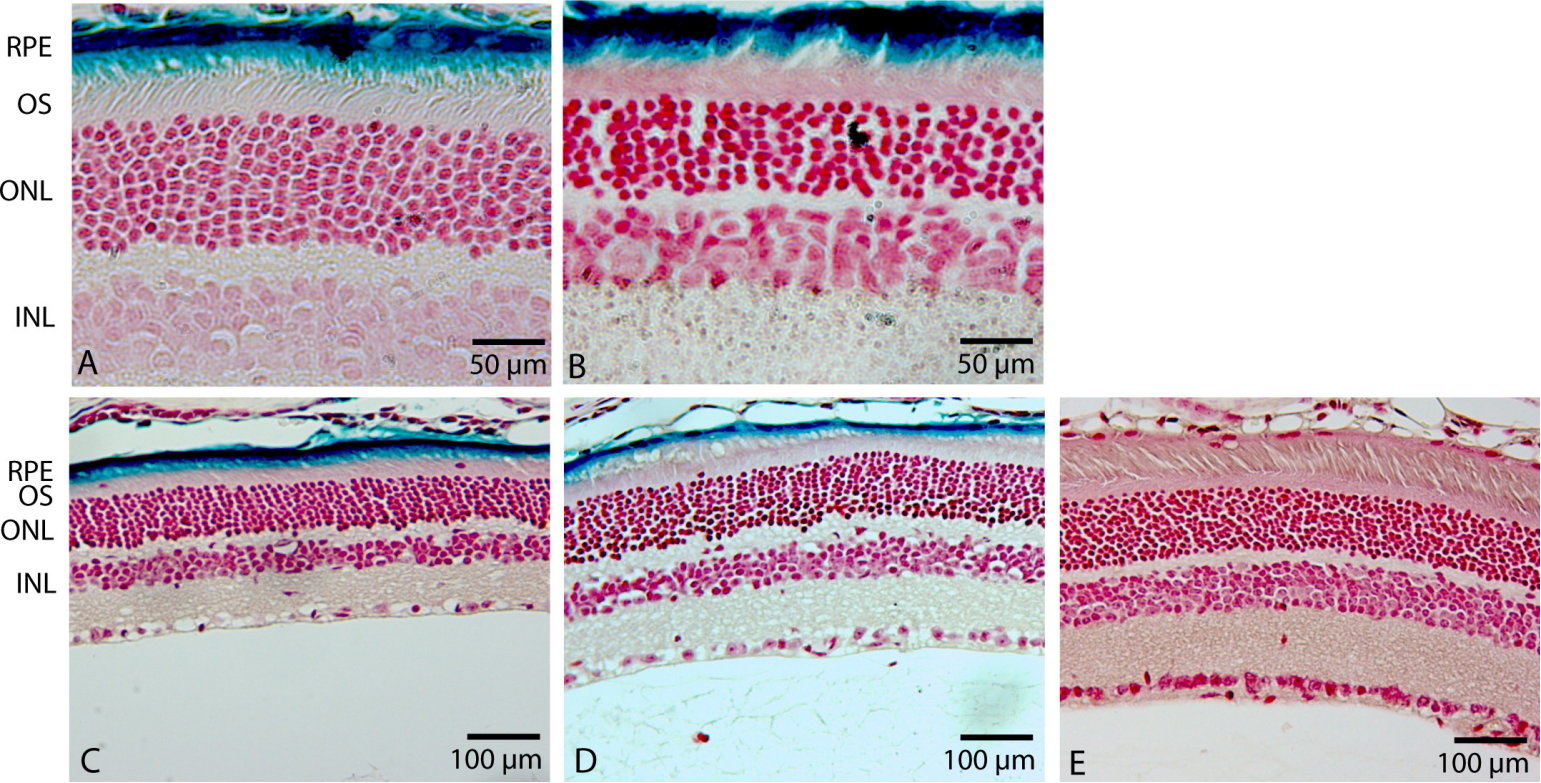
**Retinal expression of LacZ reporter**. Eyes were processed for X-gal staining and cut at serial sections of 6 μm and counterstained with neutral red. Expression was detected at 1 week (A), 2 weeks (B), 1 month (C), and 2 months (D) in the RPE layer of the retina. Some expression was also detected in the proximal OS. Control (E) with no vector injection reveals no β-galactosidase activity. RPE, retinal pigment epithelium; OS, outer segments; ONL, outer nuclear layer; INL, inner nuclear layer.

Currently, retinal gene therapy trials are carried out with AAV vectors [26] and little is known about the feasibility of HD-Ad vector as a vehicle for gene delivery to human retinal cells. To examine the potential of HD-Ad vectors to be used for retinal gene therapy, mice were given subretinal injections with the CMV-LacZ HD-Ad vector. At 4 different time points, the retinal expression of the reporter gene was determined via X-gal staining. One week following injection with HD-Ad-CMV-LacZ at 1 × 10^8 ^vector particles (vp)/eye, mice showed robust transgene expression along the retinal layer (Figure 1A). The X-gal positive areas were seen in the RPE predominantly, with sporadic expression found in the proximal regions of the outer segments (OS). Two months post-injection (Figure 1D), the furthest time point tested for the HD-Ad-CMV-LacZ injected mice, trends in expression were comparable to the 1 week time point. X-gal staining was present along the retinal layer with the majority of expression in the RPE layer and little expression in the OS of the PR cells.

HD-Ad vectors have attributes that make them desirable in gene therapy trials. Due to their genome being devoid of all viral coding genes [17,18], little or no CTL response arises [19-21], and the vector can persist in host cells for a very long time where they stay in episomal form [27]. Since retinal cells are terminally differentiated and non-replicative, dilutional loss of episomes is unlikely for HD-Ad vectors. Furthermore, since the genome is non-integrating, there is minimal risk of insertional mutagenesis [27]. Although we only examined reporter expression up to two months, we predict that it would continually persist had further time points been examined.

Transgene expression was detected in mouse retina three days following intraocular injections with HD-Ad vectors [19,25]. Our results show that onset of maximum gene expression occurred no later than 1 week post-injection. The lack of delay for transgene expression makes HD-Ad vectors superior if immediate transgene expression is desired. For example, acute damage by physical trauma to the eye resulting in fast retinal cell deterioration will require a vector that quickly delivers survival factors to rescue rapidly dying cells. The delivery of various neurotrophic factors, growth factors, and cytokines protect neurons from cell death in these instances including BDNF, CNTF, neurotrophin-3 and -4, and bFGF [28-31].

**Figure 2:**
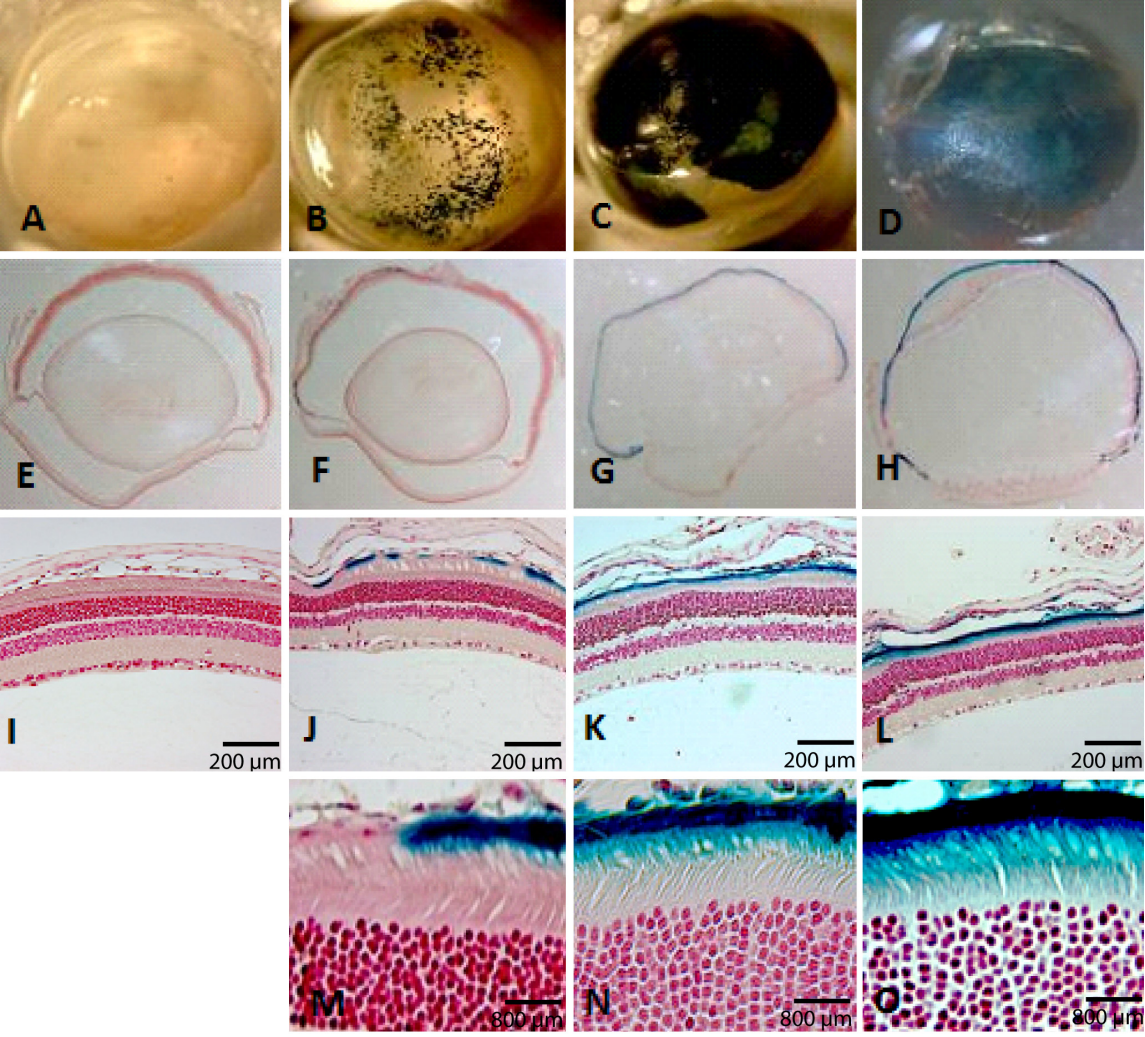
**Correlation of transgene expression with viral vector concentration**. Mouse eyes were injected with 1 μL of a CMV LacZ vector and enucleated at 2 weeks and processed for X-gal staining (B-D). Serial sections were cut at 6 μm and tissues were counterstained with neutral red to reveal retinal layers (F-H). Controls with no vector injection (A, E, I) reveal no sign of β-galactosidase activity. Eyes injected at 1 × 10^7 ^vp/μL (B, F, J) showed transgene expression to be present, but sporadic in the retina and confined to the RPE (M). At a higher dose of 1 × 10^8 ^vp/μL (C, G, K) expression was seen around the entire retinal layer and detected in the RPE predominantly (N). At 1 × 10^9 ^vp/μL (D, H, L) significant expression was detected around the entire retinal layer with staining in both the RPE layer as well as PR segments (O).

To examine the effect of viral vector dosage on transgene expression, we injected mice with 3 different viral vector doses and evaluated reporter gene expression levels. At the lowest vector dose of HD-Ad-CMV-LacZ, 1 × 10^7 ^vp/eye, transgene expression was detected (Figure 2B), but consistency of expression throughout the entire retina was not observed as only several areas around the retina showed β-galactosidase activity. Serial sectioning of tissues at this dose revealed that expression was absent from a large proportion of the retina (Figure 2F). Also, expression of the transgene was confined to the RPE layer of the retina (Figure 2M) and absent from PR cells.

At 1 × 10^8 ^vp/eye, significant LacZ expression was observed throughout the retina (Figure 2C and 2G). Serial sectioning of tissues in this group reveals consistent and widespread X-gal staining all along the RPE layer (Figure 2G and 2N). The highest vector concentration, 1 × 10^9 ^vp/eye resulted in more wide-spread X-gal staining (Figure 2H) without affecting the histology (Figure 2D). Expression at this dose revealed robust staining in the RPE as well as in photoreceptor inner and outer segments (Figure 2O).

**Figure 3:**
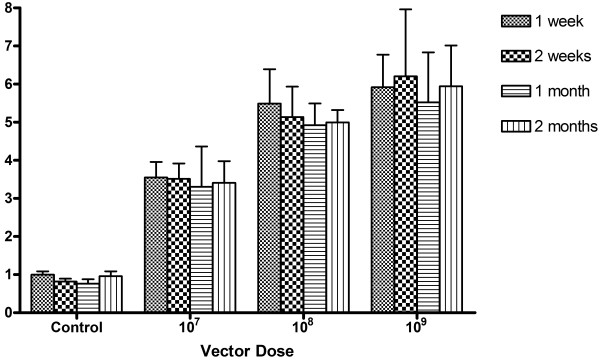
**Quantitative analysis of vector dose-dependent transgene expression**. Mouse eyes were injected with 1 μL of a CMV LacZ vector at increasing concentrations of 1 × 10^7 ^vp/μL, 1 × 10^8 ^vp/μL and 1 × 10^9 ^vp/μL. Eyes were enucleated, processed for X-gal staining and measured for β-galactosidase activity levels. Transgene expression was determined by luminescence and reported as a fold increase relative to the control. Statistical analysis revealed no significant difference between the time points. However, activity levels were determined to be significantly different with the increase in viral vector dose suggesting that a dose-dependent relationship exists (2-way ANOVA; dose p < 0.05; time p > 0.05; interaction p>0.05). Error bars indicate standard error of the mean (SEM) and 4 mice were used for each time point of each vector dose.

As a means to quantitatively evaluate the amount of reporter gene activity with viral dose, we performed a β-galactosidase activity assay. We found that with an increase in vector delivery, there was a statistically significant increase in β-galactosidase activity (Figure 3). Correspondingly, each vector dose group had a statistically significant difference in transgene activity from all other vector dose groups (1-way ANOVA and Bonferroni corrected pair-wise t-tests, p < 0.05). Additionally, at each of the vector dose groups, β-galactosidase activity levels were also measured over different time points and we found that overall there was no significant association between time points and transgene activity levels (2-way ANOVA; dose p < 0.05; time p > 0.05; interaction p > 0.05). These results demonstrate that there is a significant correlation between viral vector dosage and transgene expression.

Our results demonstrate that the viral vector delivery resulted in a dose response trend in levels of the expression, as determined by histochemical (Figure 2) and quantitative analyses (Figure 3). These results suggest that the vector dose can be used to control the level of transgene expression. Another way to regulate the amount of transgene expression is to use cis-acting DNA elements and promoters. AAV vectors are generally unable to carry these large regulatory sequences, but HD-Ad vectors with their large 37 kb cloning capacity can house multiple transgenes and native regulatory elements that promote desirable gene expression. Specifically, future studies will be directed to examine the efficiency of HD-Ad vectors for targeting transgene expression to photoreceptor cells using cell-specific promoters.

**Figure 4:**
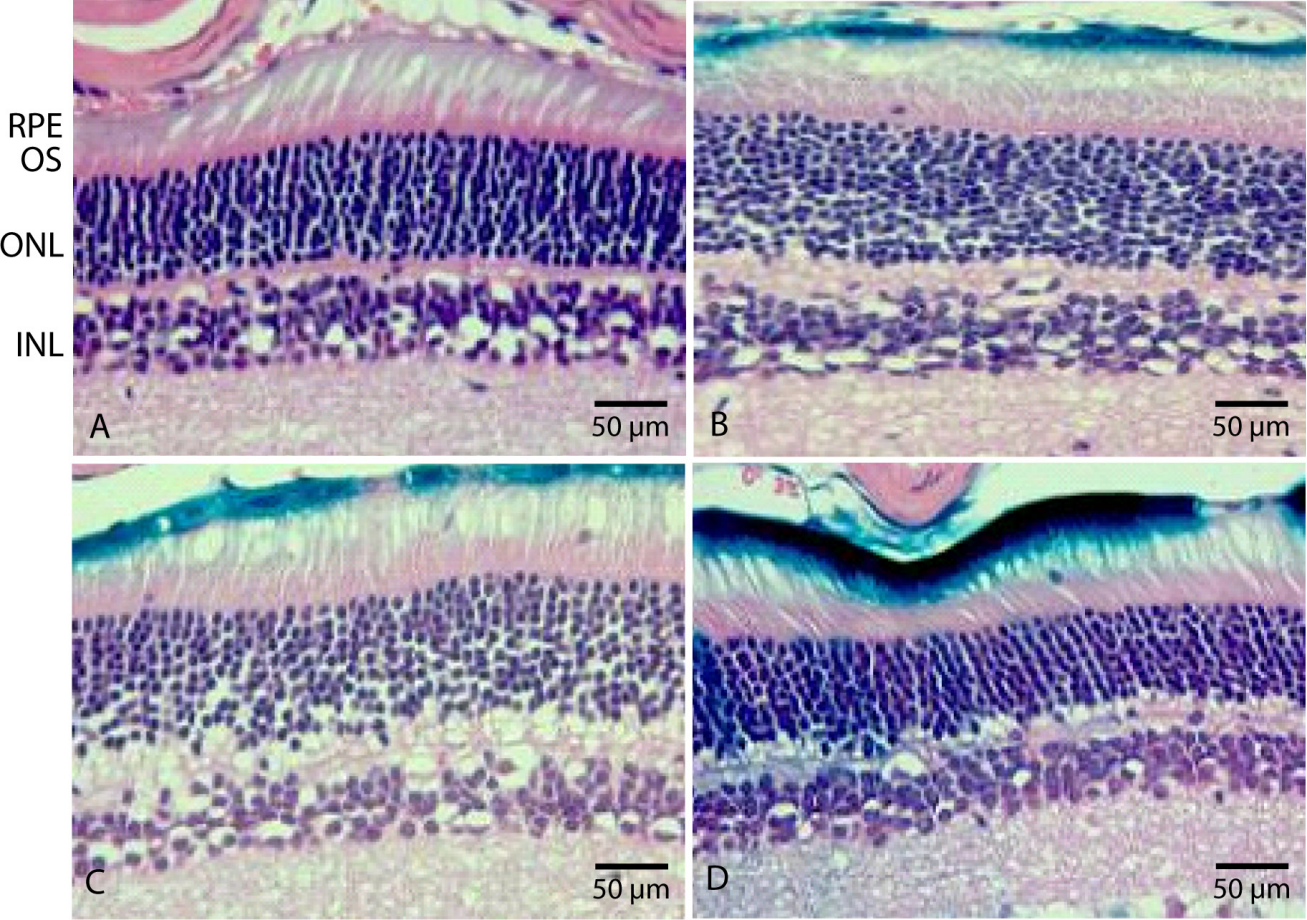
**H&E staining of retinal tissue sections**. Eyes were processed for X-gal staining at 3 different concentrations of viral vector dose at 1 week post-injection and counterstained with H&E. Controls (A) with no injection revealed no β-galactosidase activity and normal physiology. CMV LacZ injection at 1 × 10^7 ^vp/μL (B), 1 × 10^8 ^vp/μL (C), and 1 × 10^9 ^vp/μL (D) all show signs of transgene expression in the RPE layer, without visible signs of inflammation or cell toxicity. RPE retinal pigment epithelium; OS outer segments; ONL outer nuclear layer; INL inner nuclear layer.

Since potential tissue damage with high viral load is a concern for retinal gene therapy, it is important to determine the lowest amount of vector required for maximum gene expression. To examine whether HD-Ad vector delivery could cause tissue damage, we performed H&E staining of the retinas from mice that received different doses of HD-Ad-CMV-LacZ. The eyes were first processed for X-gal staining at 1 week post-injection to verify that viral delivery was successful. The X-gal stained retinal tissues were then used for H&E staining. The results showed that at three increasing viral vector concentrations, there was no visible sign of inflammation (retinal folding, granulations, cell death, and tissue necrosis) (Figure 4A and 4B and 4C). Likewise, tissue morphology of all injected mice were similar to their negative, no-injection controls (Figure 4D), suggesting that at the doses we selected, the viral vector concentration does not cause tissue damage. Our results show that at 1 week following gene delivery, morphology of the retina remains normal with little or no sign of tissue damage or inflammation. Even at our highest viral vector dose, no visible inflammation was detected in the ocular space, implying the absence of a strong immune response. This lack of immunogenicity will lead to prolonged transgene expression in the host.

## Conclusions

An ideal vector for retinal gene therapy needs to fulfill the following criteria. First, it must be able to target cells in the retina. Second, it must be able to circumvent the immune system from clearance of the vector as well as prevent an immune reaction that may damage ocular tissue. Third, it must be safe by avoiding insertional tumorigenesis. Finally, it must retain a relatively large cloning capacity for carrying large therapeutic genes as well as long expression control DNA elements. While great progress has been made with AAV based vectors, it remains incapable of carrying large therapeutic genes. The results of this study demonstrate that HD-Ad fulfills these requirements and has great potential for further research as a vector for retinal gene therapy.

## Methods

### HD-Ad Vectors and their production

HD-Ad-CMV-LacZ[32] used in this study expresses the LacZ reporter gene under the control of the cytomegalovirus immediate-early promoter (CMV). The reporter gene cassette was cloned into the viral vector pC4HSU [14] and the viral particles were prepared as described [14,15]

### Animal care and subretinal injection

One month old female CD-1 mice (Charles River Laboratories International) used this study were treated in strict compliance with the Association for Research in Vision and Ophthalmology (ARVO) statement on the Use of Animals in Ophthalmic and Vision Research. The animal protocol was approved the Animal Care Committee of the Hospital for Sick Children. For subretinal vector delivery, animals were anaesthetized via intraperitoneal injections of a mixture (100 μL/10 g body mass) of ketamine (20 mg/mL; Wyeth Animal Health), xylazine (2 mg/mL; Bayer HealthCare) in saline, and pupils were dilated with a topical application of a mixture of 0.2% Cyclogyl, 0.5% Mydfrin, and 0.1% Tropicamide (all from Alcon) in water for 30 seconds. Under an SZX12 dissection microscope (Olympus), a small incision was made through the cornea, adjacent to the limbus with a 301/2-gauge needle. A 33 gauge blunt-end needle (Hamilton) was then inserted through the incision with special care to avoid the lens, and was pushed through the retina to the subretinal space where the virus was injected very slowly. Each animal received 1 μl of virus in the right eye, leaving the left eye as a negative control. Partial retinal detachment was observed and recovered in a week post-injection.

### X-gal staining of whole eyeball

Eyes were enucleated and fixed with 1% formaldehyde, 0.1% glutaraldehyde, 2 mM MgCl_2_, 5 mM EGTA in 0.1 M sodium phosphate buffer, pH 7.8 for 30 minutes at 4°C with rocking. Fixed tissues were washed with 2 mM MgCl_2_, 0.01% Deoxycholate, 0.02% NP-40 in 0.1 M sodium phosphate buffer at 4°C with rocking and stained with X-gal in the wash solution containing 5 mM K_4_Fe(CN)_6_, 5 mM K_3_Fe(CN)_6 _and 40 mg/ml of dimethyl formaide) at 37°C with shaking for 3 hours. After staining, samples were washed 3 times with 70% ethanol and post-fixed with 10% formaldehyde at 4°C for 4 hours. Samples were then sent to the Pathology Department of The Hospital for Sick Children where they were embedded in paraffin blocks. 60 serial sections (6 μm thick) were then cut at the horizontal meridian and distributed on 10 slides representative of the whole eye at different levels. Light microscope images were taken on a DM IRB microscope (Leica). Four eyes were examined at 1 week, and 3 eyes at each of 2 weeks, 1 month, and 2 months. The un-injected eye of each animal was used as controls.

### Histology analysis

For H&E staining, tissues were deparaffinised and rehydrated in a series of alcohol rehydration steps. Slides were stained with hematoxalin (Poly Scientific) for 3 minutes and rinsed with deionized water. Tissues were dipped briefly in acidified ethanol (1 mL of concentrated HCl in 700 mL of 70% ethanol) to de-stain and rinsed with deionized water. Excess water was blotted from the slide before staining tissue with eosin (Poly Scientific) for 1 minute. Tissues were dehydrated in a series of alcohol dehydration steps and mounted with xylene-based mounting media, Permount (Fisher Scientific) and covered with a coverslip. For neutral red staining, tissues were deparaffinised and rehydrated in a series of alcohol rehydration steps. Slides were stained with neutral red staining solution (0.1% neutral red in 37 mM acetate solution, pH4.8) for 2 minutes and rinsed in running tap water until dye has been removed from slides. Tissues were dehydrated in a series of alcohol dehydration steps and mounted with xylene-based mounting media, Permount (Fisher Scientific) and covered with a coverslip.

### β-Galactosidase reporter assays

Eyes were enucleated and the lens and vitreous was removed under a dissection microscope, leaving only the eyecup. Tissue was homogenized in lysis buffer containing 100 mM potassium phosphate buffer pH 8.0 with 0.5 mM DTT, 10% Triton X-100 and proteinase inhibitor cocktail (Roche Diagnostics). Samples were centrifuged for 15 minutes at 12,000 RPM in 4°C. Supernatant was collected and either processed immediately or stored at -80°C. The lysate was heat inactivated at 48°C for 50 minutes and allowed to cool to room temperature. The β-galactosidase activity was measured using a chemiluminescent assay as described [33]. Four eyes were used for each time point at each dosage level (total of 64 eyes).

## List of abbreviations used

AAV: adeno-associated virus; CMV: cytomegalovirus; HD-Ad: helper-dependent adenoviral vector; FG-Ad: first generation Adenoviral; CTL: cytotoxic T lymphocyte; H&E: Haematoxylin and Eosin; RPE: retinal pigment epithelial; PR: photoreceptor; OS: outer segments; ONL: outer nuclear layer; INL: inner nuclear layer.

## Competing interests

The authors declare that they have no competing interests.

## Authors' contributions

All authors read and approved the final manuscript. LW carried out most of the experiments and participated in the manuscript preparation. SL participated in data analysis including the help of statistics and in revising the manuscript. HC and RG participated in the design of experiments as well as experimentation. RD prepared and purified the viral vectors. DV and RB participated in data analysis and assisted in the experimental design. RM assisted in the experimental design and participated in revising the manuscript. JH supervised the whole project including manuscript preparation.
